# Efficacy and safety of tirzepatide versus placebo in overweight or obese adults without diabetes: a systematic review and meta-analysis of randomized controlled trials

**DOI:** 10.1007/s11096-024-01779-x

**Published:** 2024-07-22

**Authors:** Ligang Liu, Hekai Shi, Merilyn Xie, Yuxiao Sun, Milap C. Nahata

**Affiliations:** 1 Institute of Therapeutic Innovations and Outcomes (ITIO), College of Pharmacy, The Ohio State University, 500 West 12th Ave., Columbus, OH 43210 USA; 2 Department of Bariatric and Metabolic Surgery, Fudan University Affiliated Huadong Hospital, Shanghai, China; 3 St. John’s University College of Pharmacy and Health Sciences, New York, NY USA; 4 Department of Rehabilitation Medicine, West China Hospital, Sichuan University, Chengdu, Sichuan China; 5 College of Medicine, The Ohio State University, Columbus, OH USA

**Keywords:** Efficacy, Meta-analysis, Obesity, Safety, Tirzepatide

## Abstract

**Background:**

Tirzepatide was approved to treat type 2 diabetes and obesity, but its efficacy and safety in patients without diabetes has not been investigated.

**Aim:**

This meta-analysis aimed to evaluate the efficacy and safety of tirzepatide compared to placebo in ﻿overweight or obese patients without diabetes.

**Method:**

PubMed, Embase and Cochrane were searched on January 18, 2024. Randomized controlled trials (RCTs) that used tirzepatide in ﻿overweight or obese adults without diabetes were included. Efficacy outcomes included the proportion of participants achieving weight loss targets, changes in body weight (%), body mass index (BMI), waist circumference (WC), and blood pressure (BP). Safety outcomes were commonly reported adverse events. Standardized mean differences (SMD) or odds ratios (OR) with 95% confidence intervals (CIs) were used for continuous and dichotomous outcomes, respectively.

**Results:**

Three RCTs with 3901 participants were included. Tirzepatide was associated with increased proportion of participants achieving weight loss targets, reduced body weight (SMD − 1.61, 95% CI − 2.20 to − 1.02), BMI (SMD − 2.13, 95% CI − 3.08 to − 1.18), WC (SMD − 0.91, 95% CI − 1.14 to − 0.69), and BP versus placebo. However, the risk of adverse events such as nausea (OR 4.26, 95% CI 2.60 to 3.81), vomiting (OR 8.35, 95% CI 5.19 to 13.45), and diarrhea (OR 3.57, 95% CI 2.80 to 4.57) was significantly higher for tirzepatide versus placebo.

**Conclusion:**

Tirzepatide significantly reduced weight and improved metabolic markers among ﻿overweight or obese without diabetes. However, increased adverse events highlights the need for benefits versus risks assessment before initiation and continuous monitoring.

**Supplementary Information:**

The online version contains supplementary material available at 10.1007/s11096-024-01779-x.

## Impact statements

- Tirzepatide significantly achieved targets for weight loss and reduced body weight, BMI, WC, BP and HbA1c among ﻿overweight or obese adults without diabetes, offering an important alternative to current GLP-1 receptor agonists like semaglutide and liraglutide.
- The increased risk of overall adverse events and treatment discontinuation due to adverse events would require careful patient monitoring during tirzepatide therapy and assessment of benefits versus risks with its use.
- Direct comparison between tirzepatide and GLP-1 receptor agonists are needed in future research.
- The findings of this study should guide healthcare providers in making informed treatment decisions for ﻿overweight or obese adults, highlighting the importance of considering tirzepatide as a viable option in weight management strategies.

## Introduction

Obesity remains a global concern with prevalence rate exceeding 40% worldwide [1]. It is not only a significant risk factor for various chronic diseases such as type 2 diabetes, cardiovascular diseases, sleep disorders, and certain cancers [2], but it also has significant implications for increasing overall mortality and healthcare costs [3, 4].

The multifaceted etiology of obesity makes its management particularly challenging [5]. While lifestyle interventions, including dietary modifications and increased physical activity, remain the cornerstone of obesity treatment [6], they often yield limited long-term success in weight reduction and maintenance [7]. Moreover, the pharmaceutical treatment is limited, and treatment discontinuation usually leads to weight regain [8]. Consequently, there is a growing interest in developing pharmacological options to augment weight loss efforts and mitigate obesity-related health risks.

Tirzepatide combines the actions of glucagon-like peptide-1 receptor agonists (GLP-1 RAs) and glucose-dependent insulinotropic polypeptide (GIP), and exerts significant effects on appetite, energy intake, and metabolic function, which could translate into significant weight loss and metabolic benefits [9]. The efficacy of tirzepatide has been well-documented in individuals with type 2 diabetes, where it has significantly improved glycemic control and led to substantial weight loss [10–12]. Moreover, it could be a better option than semaglutide in patients with type 2 diabetes [12]. These promising results have led to the investigation of tirzepatide as a potential therapeutic agent for overweight or obese adults without diabetes. The U.S. Food and Drug Administration (FDA) and National Institute for Health and Care Excellence (NICE) has approved the use of tirzepatide in adults with type 2 diabetes mellitus and adults with obesity [13–16]. However, the indication of tirzepatide for obesity has not been established in other countries.

Several recent randomized clinical trials (RCTs) have highlighted  the efficacy of tirzepatide in weight reduction and cardiovascular benefits among ﻿overweight or obese individuals without diabetes [17–19]. Despite the growing body of evidence supporting the use of tirzepatide in obesity, there is a need to rigorously evaluate the efficacy and safety of tirzepatide in reducing body weight and improving metabolic health in adults with obesity but without diabetes.

### Aim

This systematic review and meta-analysis aimed to evaluate the efficacy and safety of tirzepatide compared to placebo in overweight or obese adults without diabetes.

## Method

PubMed, Embase, and the Cochrane Library were comprehensively searched on January 18, 2024, to identify relevant literature. Key search terms included "tirzepatide," "LY3298176," "dual GIP and GLP-1RA," "Zepbound," and "randomized controlled trial". The detailed search strategy for each database is provided in the Supplementary File 1. This systematic review and meta-analysis protocol was prospectively registered in PROSPERO with registry number CRD42024502434. This systematic review was conducted and reported in accordance with the Preferred Reporting Items for Systematic Reviews and Meta-Analyses (PRISMA) guidelines [20].

The inclusion criteria were specified as: (1) RCTs that investigated ﻿overweight or obese adult participants without diabetes; (2) tirzepatide as the intervention; (3) any active treatment or placebo as the control; (4) reporting of at least one efficacy outcome.

### Outcomes measures

Efficacy outcomes included the proportion of patients achieving weight loss goals of 5%, 10%, 15%, 20%, or 25%, weight change, body mass index (BMI) change, waist circumference (WC) change, glycated hemoglobin (HbA1c) change, systolic blood pressure (SBP) and diastolic blood pressure (DBP) change, 36-item short-form health survey (SF-36) physical functioning score or impact of weight on quality of life (IWQOL) score change. The percentage of weight loss caused by tirzepatide was the primary efficacy outcome. Other outcomes were secondary efficacy outcomes. Safety outcomes included overall adverse events (AEs), serious adverse events (SAEs), treatment discontinuation due to adverse events, and commonly reported AEs.

Title and abstract screening, followed by full-text assessments to confirm trial eligibility, were conducted independently by two authors (L.L. and Y.S.). Any disagreements were resolved by involving a third researcher (M.N.). Data were extracted by L.L. and Y.S., including study design, sample size, therapy duration, interventions, controls, baseline characteristics, and outcome measures. The data extraction process was cross-checked by another author (M.N.) to ensure accuracy. The Cochrane Risk of Bias tool for randomized controlled trials version 2 was employed to evaluate the quality of the RCTs [21]. The assessment was independently performed by two authors (L.L. and Y.S.), and discrepancies were resolved through discussion with a third author (M.N.).

All analyses were performed using R (version 4.1.2). Generalized linear mixed models were initially performed to pool the outcomes across different trials, with mean difference (MD) between groups or percentage of patients achieving weight loss goals as the standard for pooling the results. Then, meta-analyses were conducted to compare tirzepatide versus placebo. Standardized mean difference (SMD) or odds ratio (OR) with 95% confidence intervals (CIs) were used to assess different outcomes as appropriate. In this analysis, a 95% CI that did not contain the value 0 for standardized mean differences or 1 for odds ratios indicated statistical significance (*p* < 0.05). Conversely, CIs that included these values suggested non-significance (*p* ≥ 0.05). The SMD was utilized to quantify the effect size of the intervention: an SMD of less than 0.2 was considered small, indicating a minimal practical significance; an SMD between 0.2 and 0.5 was regarded as medium, suggesting a moderate effect; and an SMD greater than 0.8 was classified as large, reflecting a substantial impact [22]. Publication biases were not assessed due to a limited number of studies. The I^2^ was used to evaluate heterogeneity among trials. An I^2^ of less than 25% indicates negligible heterogeneity, and a fixed-effect model was employed. In cases when I^2^ was above 50% (substantial heterogeneity), and a random-effect model was chosen.

## Results

**Fig. 1:**
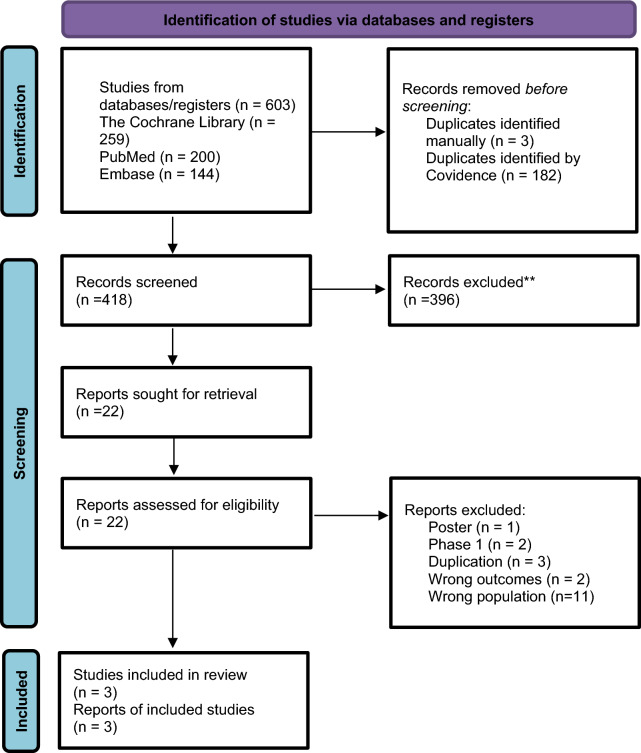
Flow diagram of study screening and selection

We identified 603 potentially relevant references. After the removal of duplicates, 418 references were retrieved. Finally, three RCTs met the inclusion criteria (Fig. 1), and 3901 participants were included in these trials [17–19]. In SURMOUNT-1 trial, participants were administered tirzepatide at doses of 5 mg, 10 mg, and 15 mg over 72 weeks. In SURMOUNT-3, participants received the maximum tolerated dose, either 10 mg or 15 mg, also for 72 weeks. Similarly, in the SURMOUNT-4 trial, participants received the maximum tolerated dose for 88 weeks. The efficacy outcomes of tirzepatide in all trials were measured at the conclusion of the respective study periods. The characteristics and efficacy results of the included studies are presented in Table 1. No risk of bias was observed, as summarized in Table S1.

**Table 1:** Characteristics and efficacy outcomes of included trials

		SURMOUNT-1 [19]	SURMOUNT-3 [18]	SURMOUNT-4 [17]		
Author		Venniyoor et al.	Wadden et al.	Aronne et al.		
Route and frequency		Subcutaneous, once weekly				
Design/Phase		Phase 3 double-blind, randomized, controlled trial	Phase 3 double-blind, randomized, controlled trial	Phase 3, randomized, double-blind, placebo-controlled		
Country		119 sites in nine countries	Argentina, Brazil, and USA	70 sites in Argentina, Brazil, Taiwan, and the US		
Comparator (# of participants)		5 mg (630) vs 10 mg (636) vs 15 mg (630) vs placebo (643)	^a^MTD (287) vs placebo (292)	^a^MTD (335) vs Placebo (335)		
Mean age (SD)		44.9 (12.5)	45.6(12.2)	48 (13)		
Gender (female), %		67.5	62.9	70.6		
Trial duration (weeks)		72	72	0–36	36–88	0–88
*Efficacy outcomes*						
WL, %	TRE	− 15.0 vs. − 19.5 vs. − 20.9 vs. − 3.1	− 18.4 vs. 2.5	− 21.1 vs. − 20.8	− 5.5 vs. 14.0	NA
	EE	− 16.0 vs. − 21.4 vs. − 22.5 vs. − 2.4	− 21.1 vs. 3.3		− 6.7 vs. 14.8	− 25.8 vs − 9.5
≥ 5% WL, %	TRE	85.1 vs. 88.9 vs. 90.9 vs. 34.5	87.5 vs. 16.5	NA		97.3 vs. 70.3
	EE	89.4 vs. 96.2 vs. 96.3 vs. 27.9	94.4 vs. 10.7	NA		98.5 vs. 69.0
≥ 10% WL, %	TRE	68.5 vs. 78.1vs. 83.5 vs. 18.8	76.7 vs. 8.9	NA		92.1 vs. 46.2
	EE	73.4 vs. 85.9 vs. 90.1vs. 13.5	88.0 vs. 4.8	NA		94.0 vs. 44.4
≥ 15% WL, %	TRE	48.0 vs. 66.6 vs. 70.6 vs. 8.8	65.4 vs. 4.2	NA		84.1 vs. 25.9
	EE	50.2 vs. 73.6 vs. 78.2 vs. 6.0	73.9 vs. 2.1	NA		87.1 vs. 24.0
≥ 20% WL, %	TRE	30.0 vs. 50.1 vs. 56.7 vs. 3.1	44.7 vs. 2.2	NA		69.5 vs. 12.6
	EE	31.6 vs. 55.5 vs. 62.9 vs. 1.3	54.9 vs. 1.0	NA		72.6 vs. 11.6
≥ 25% WL, %	TRE	15.3 vs. 32.3 vs. 36.2 vs. 1.5	28.7 vs. 1.2	NA		54.5 vs. 5.0
	EE	16.5 vs. 35.0 vs. 39.7 vs. 0.3	36.3 vs. 0.3	NA		56.6 vs. 4.0
Change in WC, cm	TRE	− 14.0 vs. − 17.7 vs. − 18.5 vs. − 4.0	− 14.6 vs. 0.2	− 18.2 vs. − 17.4	− 4.3 vs. 7.8	− 22.5 vs − 9.3
	EE	− 14.6 vs. − 19.4 vs. − 19.9 vs. − 3.4	NA		− 4.6 vs. 8.3	− 22.8 vs − 9.1
^b^ Change in SBP, mmHg		− 7.0 vs − 8.2 vs − 7.6 vs − 1.2	− 5.1 vs. 4.1	− 11.8 vs. − 10.5	2.1 vs. 8.4	− 9.3 vs. − 2.4
^b^ Change in DBP, mmHg		− 5.2 vs − 5.5 vs − 4.6 vs − 1.0	− 3.2 vs. 2.3	− 5.4 vs. − 4.9	− 0.4 vs. 3.2	− 5.5 vs. − 1.7
^b^ Change in TC		− 4.9 vs − 5.6 vs − 7.4 vs − 1.1	− 3.0 vs. 5.2	− 5.6 vs. − 4.8	2.3 vs. 8.3	− 5.0 vs. 2.2
^b^ Change in TG		− 24.3 vs − 27.0 vs − 31.4 vs − 6.3	− 25.8 vs. 3.0	− 23.1 vs. − 21.2	− 8.2 vs. 15.6	− 33.3 vs. − 15.3
^b^ Change in HbA1c		− 0.40 vs − 0.49 vs − 0.51 vs − 0.07	− 0.5 vs. 0.0	− 0.5 vs. − 0.5	− 0.08 vs. 0.25	− 0.57 vs. − 0.22
^b^ Change in BMI		NA	− 7.7 vs. 1.2	− 8.0 vs. − 7.9	− 2.1 vs. 4.3	− 10.0 vs. − 3.6
^b^SF-36v2 PF Score		3.9 vs 3.9 vs 4.2 vs 1.9	3.3 vs. − 0.6	5.9 vs. 5.5	0.8 vs. − 1.8	6.4 vs. 3.7
^b^ IWQOL Score		NA	13.9 vs. 1.1	22.0 vs. 22.2	4.3 vs. − 5.1	26.0 vs. 16.7

*MTD* maximum tolerated dose, *WL* weight loss, *BMI* body mass index, *WC* waist circumference, *HbA1c* hemoglobin A1c, *SBP* systolic blood pressure, *DBP* diastolic blood pressure, *TC* total cholesterol, *TG* triglycerides, *SF-36 PF* short form-36 health survey physical functioning domain, *IWQOL* impact of weight on quality of life, *TRE* treatment-regimen estimand, *EE* Efficacy estimand, *AE* adverse events, *SAE* serious adverse events, *NA* not available

^a^Tirzepatide maximum tolerated dose in SURMOUNT-3 and SURMOUNT-4 was 10 or 15 mg

^b^Change in SBP, DBP, TC, TG, HbA1c, BMI, SF-36v2 PF score, IWQOL score were presented using efficacy estimand

The pooled analysis showed that the tirzepatide led to a significant weight loss of 18.7% (95% CI − 21.3% to − 16.2%) versus placebo. The percentages of patients achieving weight loss of ≥ 5%, 10%, 15%, 20%, and 25% were 92.7%, 84.4%, 73.6%, 56.1%, and 38.7%, respectively. BMI reduction also favored tirzepatide over placebo (MD: − 7.65 kg/m^2^, 95% CI − 10.10 to − 5.20). The pooled reduction in waist circumference with tirzepatide was 14 cm (95% CI − 14.94 to − 13.06) compared to placebo. Furthermore, significant improvements were observed in both systolic and diastolic blood pressures, with reductions of 7.37 mmHg (95% CI: − 9.12 to − 5.62) and 4.38 mmHg (95% CI: − 5.39 to − 3.36), respectively. Additionally, tirzepatide showed a substantial reduction in HbA1c (MD: − 0.4%, 95% CI − 0.6% to − 0.3%) and significantly improved IWQOL score (MD: 10.95, 95% CI 7.53 to 14.38) and SF-36 score (MD: 2.87, 95% CI 1.73 to 4.00) compared to placebo (Figure S1; Table 2).

**Table 2:** Pooled efficacy and meta-analysis results of tirzepatide versus placebo

Outcomes	Pooled results		Tirzepatide vs. placebo	
	Value	95% CI	OR	95% CI
5% weight loss (%)	92.7	[85.2; 96.5]	21.14	[12.64; 35.35]
10% weight loss (%)	84.4	[74.5; 90.9]	20.03	[12.47; 32.17]
15% weight loss (%)	73.6	[62.7; 82.2]	23.61	[13.46; 41.41]
20% weight loss (%)	56.1	[44.1; 67.5]	26.52	[14.69; 47.88]
25% weight loss (%)	38.7	[27.1; 51.6]	22.75	[13.39; 38.65]

	Value	95% CI	SMD	95% CI
WL (%):	− 18.73	[− 21.31; − 16.15]	− 1.61	[− 2.20; − 1.02]
BMI change (kg/m^2^)	− 7.65	[− 10.10; − 5.20]	− 2.13	[− 3.08; − 1.18]
WC Change (cm)	− 14	[− 14.94; − 13.06]	− 0.91	[− 1.14; − 0.69]
HbA1c change (%)	− 0.42	[− 0.58; − 0.26]	− 0.77	[− 0.92; − 0.61]
SBP change (mmHg)	− 7.37	[− 9.12; − 5.62]	− 0.94	[− 1.63; − 0.24]
DBP change (mmHg)	− 7.08	[− 9.18; − 4.30]	− 0.52	[− 0.61; − 0.43]
SF-36 PF score change	2.87	[1.73; 4.00]	0.35	[0.13; 0.57]
IWQOL score change	10.95	[7.53; 14.38]	0.51	[0.23; 0.79]

*WL* weight loss, *BMI* body mass index, *SBP* systolic blood pressure, *DBP* diastolic blood pressure, *WC* waist circumference, *HbA1c* hemoglobin A1c, *SF-36 PF* short form-36 health survey physical functioning domain, *IWQOL* impact of weight on quality of life, *SMD* standardized mean difference, *OR* odds ratio, *CI* confidence interval

**Fig. 2:**
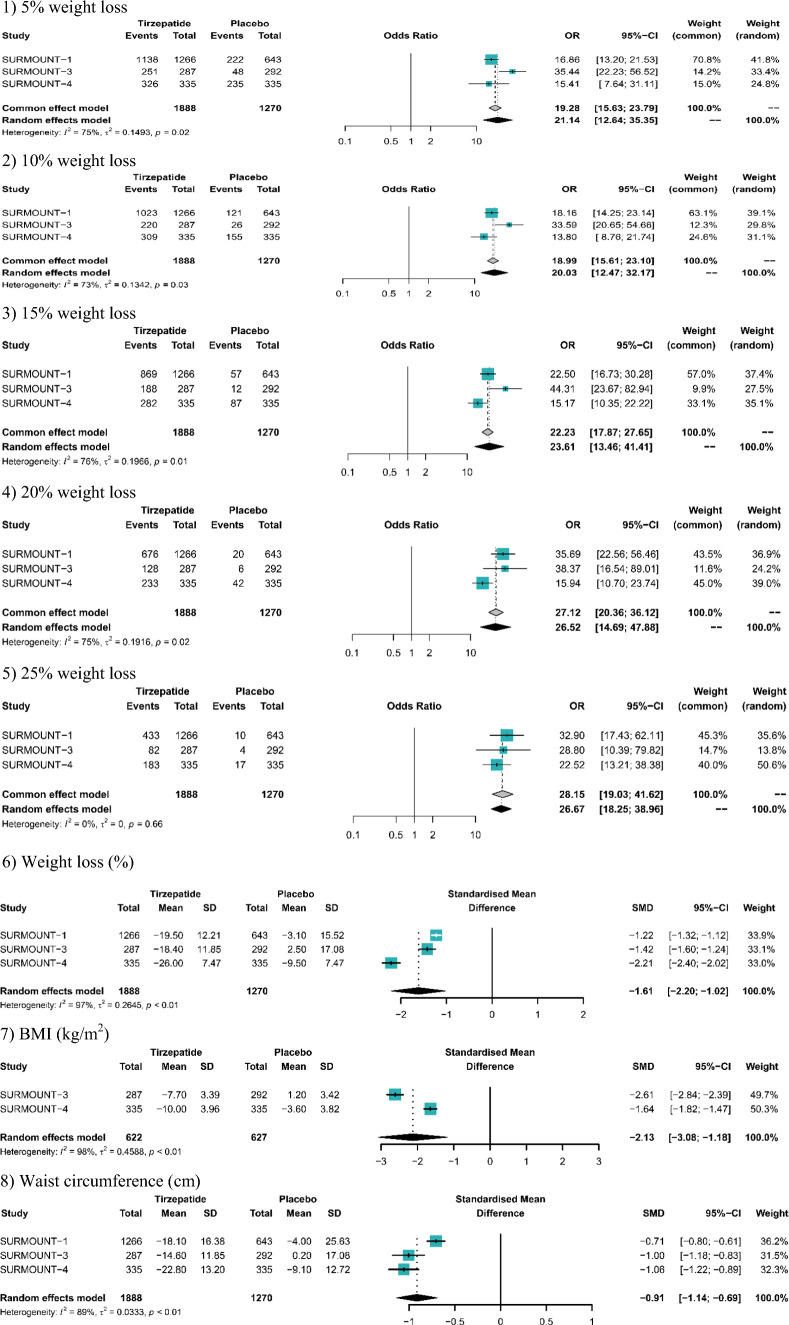
Comparison of tirzepatide versus placebo for main efficacy outcomes: (1) 5% weight loss; (2) 10% weight loss; (3) 15% weight loss; (4) 20% weight loss; (5) 25% weight loss; (6) Weight loss (%); (7) BMI (kg/m^2^); (8) Waist circumference (cm). *BMI* body mass index, *SMD* standardized mean difference, *OR* odds ratio, *CI* confidence interval

Patients prescribed tirzepatide had more significant weight loss than placebo (SMD: − 1.61, 95% CI − 2.20 to − 1.02). Participants in the tirzepatide group demonstrated a significantly higher likelihood of achieving weight loss targets of 5%, 10%, 15%, 20%, and 25% compared to those receiving placebo. Similarly, the decrease in BMI from baseline favored tirzepatide over placebo (SMD: − 2.13, 95% CI − 3.08 to − 1.18). Furthermore, tirzepatide significantly reduced waist circumference from baseline compared to placebo (SMD: − 0.91, 95% CI − 1.14 to − 0.69) (Table 2; Fig. 2). Tirzepatide also significantly reduced SBP (SMD: − 0.75, 95% CI − 0.83 to − 0.68) and DBP (SMD: − 0.51, 95% CI − 0.59 to − 0.44) versus placebo (Table 2; Figure S2). SF-36 physical function score (SMD: 0.35, 95% CI 0.13 to 0.57) and IWQOL score (SMD: 0.51, 95% CI 0.23 to 0.79) also showed significant improvement with tirzepatide compared to placebo (Figure S2).

**Fig. 3:**
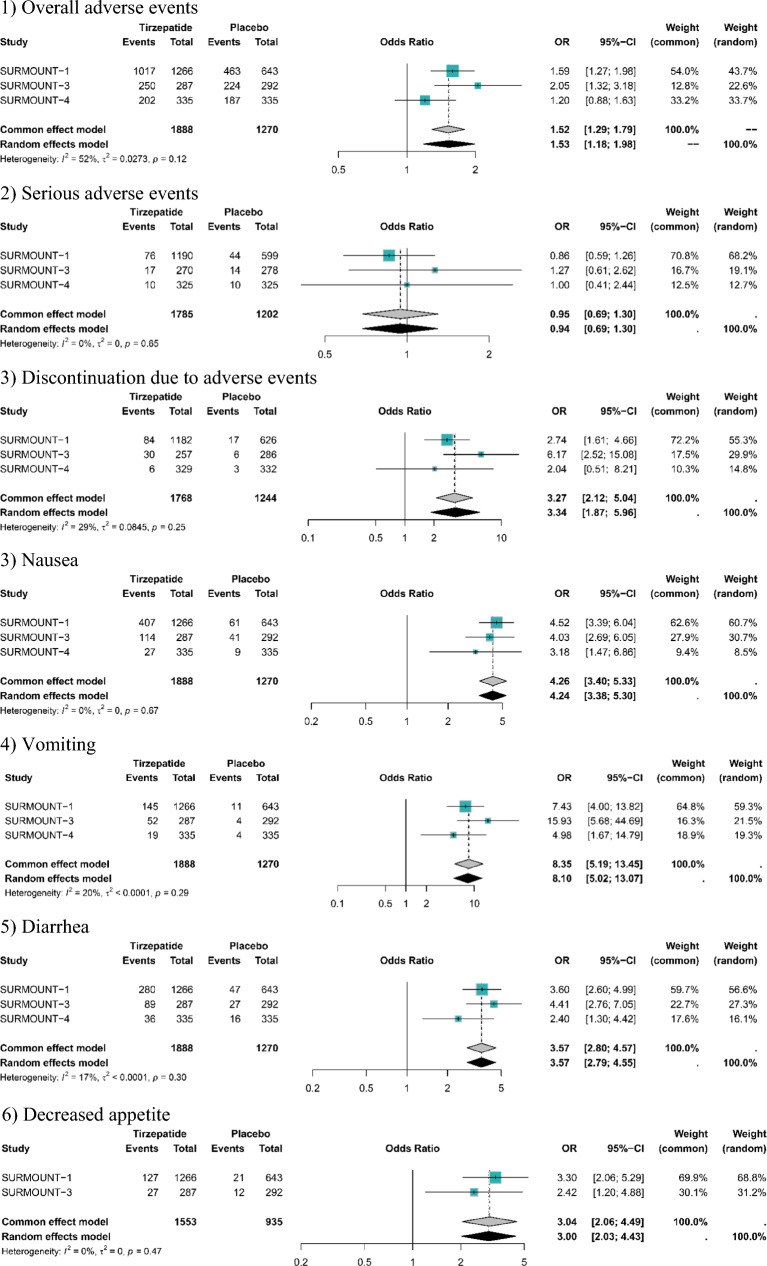

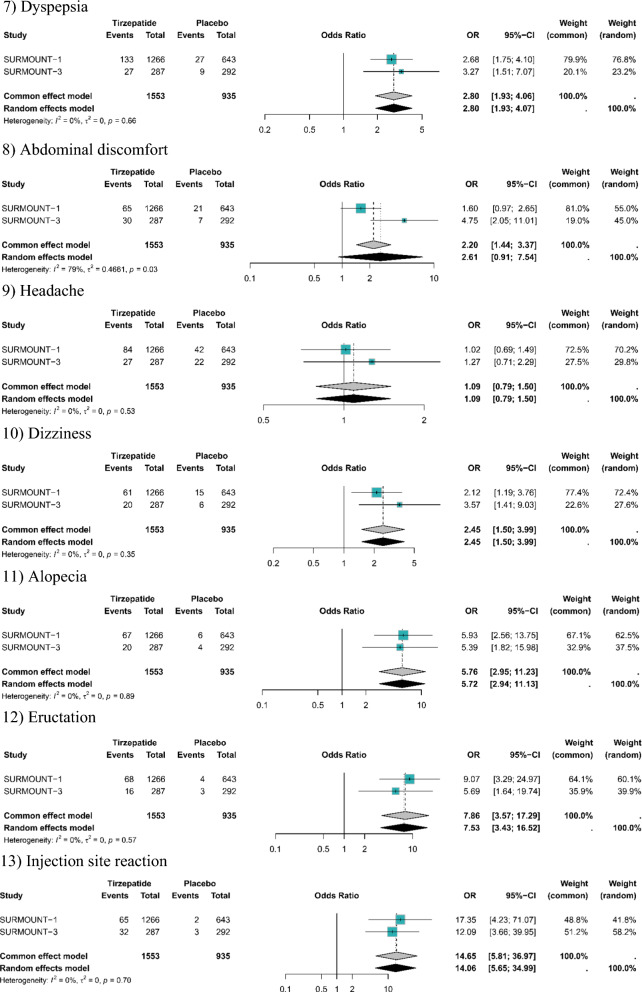
Comparison of tirzepatide versus placebo for safety outcomes: (1) Overall adverse events; (2) Serious adverse events; (3) Discontinuation due to adverse events; (3) Nausea; (4) Vomiting; (5) Diarrhea; (6) Decreased appetite; (7) Dyspepsia; (8) Abdominal discomfort; (9) Headache; (10) Dizziness; (11) Alopecia; (12) Eructation; (13) Injection site reaction. *OR* odds ratio, *CI* confidence interval

Compared with placebo, tirzepatide did not significantly increase the risk of serious adverse events (OR: 0.95, 95% CI: 0.69 to 1.30). However, the risk of overall adverse events (OR: 1.53, 95% CI: 1.18 to 1.98) and treatment discontinuation induced by adverse events (OR: 3.27, 95% CI: 3.40 to 5.33) was significantly higher among patients who received tirzepatide than placebo. As for the specific adverse events, tirzepatide had a higher risk of nausea (OR: 4.26, 95% CI: 2.60 to 3.81), vomiting (OR: 8.35, 95% CI: 5.19 to 13.45), diarrhea (OR: 3.57, 95% CI: 2.80 to 4.57), decreased appetite (OR: 3.04, 95% CI: 2.06 to 4.49), dyspepsia (OR: 2.8, 95% CI: 1.93 to 4.06), dizziness (OR: 2.45, 95% CI: 1.50 to 3.99), injection site reaction (OR: 14.65, 95% CI: 5.81; 31.70), eructation (OR: 7.86, 95% CI: 3.57 to 36.97), and alopecia (OR: 5.76, 95% CI: 2.95 to 11.23) versus placebo. However, the risk of headache (OR: 1.08, 95% CI: 0.80 to 1.46) and abdominal discomfort (OR: 2.61, 95% CI: 0.91 to 7.54) were not significantly increased with tirzepatide compared to placebo (Table 3; Fig. 3).

**Table 3:** Results for the safety profile of tirzepatide versus placebo

	Tirzepatide vs. placebo				
Outcomes	SURMOUNT-1	SURMOUNT-3	SURMOUNT-4	Pooled results	
	N (%)	N (%)	N (%)	OR	95% CI
Serious adverse events	116 (6.1%) vs. 44 (6.8%)	17 (5.9%) vs. 14 (4.8%)	10 (3.0%) vs. 10 (3.0%)	0.95	[0.69; 1.30]
Overall adverse events	1527 (80.5%) vs. 463 (72.0%)	250 (87.1%) vs. 224 (76.7%)	202 (60%) vs. 187 (56%)	1.53	[1.18; 1.98]
AEs leading to DC	111 (5.9%) vs. 17 (2.6%)	30 (10.5%) vs. 6 (2.1%)	6 (1.8%) vs. 3 (0.9%)	3.27	[3.40; 5.33]
Nausea	562 (29.6%) vs. 61 (9.5%)	114 (39.7%) vs. 41 (14.0%)	27 (8.1%) vs. 9 (2.7%)	4.26	[2.60; 3.81]
Vomiting	197 (10.4%) vs. 11 (1.7%)	52 (18.1%) vs. 4 (1.4%)	19 (5.7%) vs. 4 (1.2%)	8.35	[5.19; 13.45]
Diarrhea	398 (21.0%) vs. 47 (7.3%)	89 (31.0%) vs. 27 (9.2%)	36 (10.7%) vs. 16 (4.8%)	3.57	[2.80; 4.57]
Decreased appetite	186 (9.8%) vs. 21 (3.3%)	27 (9.4%) vs. 12 (4.1%)	NA	3.04	[2.06; 4.49]
Dyspepsia	189 (10.0%) vs. 27 (4.2%)	27 (9.4%) vs. 9 (3.1%)	NA	2.8	[1.93; 4.06]
Dizziness	87 (4.6%) vs. 15 (2.3%)	20 (7.0%) vs. 6 (2.1%)	NA	2.45	[1.50; 3.99]
Injection site reaction	83 (4.4%) vs. 2 (0.3%)	32 (11.1%) vs. 3 (1.0%)	NA	14.65	[5.81; 31.70]
Eructation	92 (4.9%) vs. 4 (0.6%)	16 (5.6%) vs. 3 (1.0%)	NA	7.86	[3.57; 36.97]
Alopecia	99 (5.2%) vs. 6 (0.9%)	20 (7.0%) vs. 4 (1.4%)	NA	5.76	[2.95; 11.23]
Abdominal discomfort	96 (5.1%) vs. 21 (3.3%)	30 (10.5%) vs. 7 (2.4%)	NA	2.61	[0.91; 7.54]
Headache	125 (6.6%) vs. 42 (6.5%)	27 (9.4%) vs. 22 (7.5%)	NA	1.09	[0.79; 1.50]

*AEs* adverse events, *DC* discontinuation, *N* number, *NA* not available, *OR* odds ratio, *CI* confidence interval

## Discussion

### Statement of key findings

We performed a meta-analysis to assess the efficacy and safety of tirzepatide in overweight or obese adults without diabetes based on data from three RCTs. The findings confirmed that tirzepatide demonstrated a significant weight loss effect compared to placebo. In summary, tirzepatide significantly decreased weight by 18.7%, BMI by 7.65 kg/m^2^, and WC by 14 cm compared to placebo. Moreover, it markedly increased the proportion of participants achieving weight reductions of 5%, 10%, 15%, 20%, and 25% versus placebo. Importantly, tirzepatide also significantly reduced HbA1c as well as systolic and diastolic blood pressure, highlighting its potential to decrease cardiovascular risk factors associated with excess weight. Improvements were also noted in the IWQOL-Lite and SF-36 physical functioning scores during treatment. However, the incidence of adverse events, particularly gastrointestinal effects such as nausea, vomiting, and diarrhea, was higher in the tirzepatide group compared to placebo. The risk of treatment discontinuation due to adverse effects also increased with tirzepatide. These adverse events raise concerns about the tolerability of tirzepatide, potentially limiting its acceptability among certain patients and emphasizing a need for monitoring therapy.

### Strengths and weaknesses

This review represents the first systematic review and meta-analysis of evidence from RCTs evaluating the efficacy and safety of tirzepatide in  ﻿overweight or obese patients without diabetes. Additionally, the comprehensive nature of the efficacy outcomes such as weight loss, reduction in BMI, waist circumference, A1c, blood pressure, and quality of life improvement provided a holistic view of efficacy profile of tirzepatide. The individual assessment of different adverse events, serious adverse events, and discontinuation due to adverse events allowed for a nuanced understanding of the safety profile of tirzepatide. However, it is important to acknowledge some limitations of this study. To date, only three RCTs have been conducted in this population, all comparing tirzepatide to placebo. The relatively small number of included trials may limit the scope of the analysis, and publication bias was not assessed due to the limited number of studies. The dose-dependence effects of tripeptide were not analyzed due to methodological differences between trials.

### Interpretation and further research

Obesity is a global health concern with negative implications for overall well-being [23, 24]. The prevalence has soared internationally and nearly tripled since 1975, according to the World Health Organization (WHO) [25]. In 2016, over 650 million adults had obesity, and it is estimated that approximately 167 million additional people will become overweight or obese by 2025 [26]. Despite the existence of numerous weight management strategies, effective treatment with sustained efficacy and safety remains a challenge.

Current FDA-approved GLP-1 RAs and its analogues for obesity include liraglutide, semaglutide, and tirzepatide [15, 27]. Previous meta-analyses have demonstrated that semaglutide can significantly reduce weight (MD: − 10.1% to − 11.8%), BMI (MD: − 3.7 to − 4.5 kg/m^2^), and WC (MD: − 8.28 to − 9.4 cm), with a higher proportion of participants achieving weight loss targets of more than 5%, 10%, 15%, and 20% [28, 29]. Liraglutide also can lead to a significant reduction in body weight (MD: − 5.04 kg), BMI (MD: − 1.95 kg/m^2^), and waist circumference (MD: − 3.64 cm), with a higher percentage of participants achieving 5% and 10% weight loss [30].

The SURMOUNT clinical trials encompassed a sequence of studies designed to assess the efficacy and safety of tirzepatide in individuals classified as overweight or obese [17–19]. SURMOUNT-1 enrolled participants with obesity or overweight and examined the weight reduction effects of tirzepatide over a 72-week main phase [19]. SURMOUNT-3 introduced a 12-week intensive lifestyle intervention before administering tirzepatide. Over an extended duration of 84 weeks, participants experienced a substantial mean weight loss of 21.1% subsequent to the lifestyle intervention phase [18]. SURMOUNT-4 implemented a 36-week lead-in period with tirzepatide treatment, followed by an additional 52 weeks of ongoing tirzepatide therapy, resulting in a 25.8% weight loss [17]. Each of these studies contributes to a comprehensive understanding of the potential of tirzepatide as a transformative treatment for obesity, highlighting not only its direct effects but also the enhanced benefits when combined with lifestyle modifications.

Recent studies have underscored the excellent efficacy of tirzepatide in promoting significant weight loss compared to semaglutide and liraglutide. Tirzepatide consistently shows a higher likelihood of achieving substantial weight reduction targets. The odds ratio of reaching a weight loss of ≥ 5% was significantly higher with tirzepatide (OR = 21.14) compared to semaglutide (OR = 2.24) and liraglutide (OR = 2.21). Similarly, the benchmarks for 10%, 15%, and ≥ 20% weight loss further illustrate robust performance of tirzepatide (10%: OR = 20.03; 15%: OR = 23.61; ≥ 20%: OR = 26.52) against semaglutide (10%: OR = 4.17; 15%: OR = 7.05; ≥ 20%: OR = 11.85) and liraglutide (10%: OR = 3.36) based on the aggregate data from our research and key prior studies [28–30]. In clinical practice, weight loss exceeding 10% from baseline is considered an excellent response [31]. The weight loss caused by tirzepatide was 18.73%, which could be clinically significant. In adults with diabetes with or without obesity/overweight, tirzepatide significantly reduced body weight (MD: 11.8% to 12.4%), BMI (MD: − 3.9 kg/m^2^), and waist circumference (MD: − 9.2 cm) [32]. Tirzepatide was more effective than semaglutide in patients with diabetes who were obese or overweight [33]. Preliminary data from our study support the hypothesis that tirzepatide may lead to superior outcomes in weight reduction, BMI, and waist circumference compared to semaglutide and liraglutide in non-diabetic obese individuals. Alkhezi et al. also reported that tirzepatide was associated with greater efficacy while maintaining a safety profile comparable to semaglutide and liraglutide [34]. However, head-to-head randomized clinical trials are needed to confirm this hypothesis further.

Despite the potential benefits offered by tirzepatide, the significantly increased risk of adverse events was a concern for the treatment of patients with obesity. The higher risk of adverse events could be a barrier to medication adherence, and these side effects must be balanced against the benefits of treatment. In clinical trials, the most reported adverse events included nausea, vomiting, diarrhea, decreased appetite, dyspepsia, dizziness, and injection site injection [17–19]. Tirzepatide had a higher risk of gastrointestinal adverse events such as nausea, vomiting, diarrhea, dyspepsia, and decreased appetite than placebo based on our findings. Previous studies also reported an increased risk of gastrointestinal adverse events with tripeptide versus placebo in patients with type 2 diabetes [32, 35]. Moreover, we also observed an increased risk of injection site reaction. Injection-associated complications also can potentially reduce patient adherence [36]. Therefore, the safety profile of tirzepatide should be considered when prescribing it to patients.

Given the increased risk of gastrointestinal adverse events associated with tirzepatide, clinicians must be diligent when prescribing this medication. The frequent occurrence of adverse events like nausea and diarrhea could significantly affect the daily activities of patients and their willingness to continue with therapy. The patient selection process for tirzepatide therapy should also be strategic, with careful consideration given to the patient’s previous history of gastrointestinal sensitivity and their ability to tolerate similar therapies. This stratification could help to minimize the risk of adverse events and improve the overall tolerability profile of the medication. An important approach to minimize the possibility of adverse events could be to start treatment at the lowest tirzepatide dose and increase it gradually as needed to achieve desired efficacy at the lowest effective dose. Furthermore, the observed increase in injection site reactions raises concerns about the administration technique and formulation of tirzepatide. This highlights the need for improved injection devices and patient training in administration techniques. We also observed the increased risk of alopecia in tirzepatide group, possibly due to nutrient deficiencies and hormonal changes caused by rapid and significant weight loss [37].

As for the scheduling for administration, tirzepatide and semaglutide are administered once weekly, while liraglutide is given once daily. For injectable medications, products administered on a once-weekly basis may provide the benefit of improved adherence and greater ease of use versus once-daily treatments [38]. Therefore, once-weekly tirzepatide might offer a more convenient and patient-friendly option compared to daily liraglutide, potentially leading to greater adherence and treatment outcomes. The less frequent dosing schedule may reduce the burden on patients, making it easier to integrate the medication into their routine and maintain consistent use over time.

Future research should directly compare tirzepatide with other active treatments in this population and investigate adverse events to provide a robust understanding of its efficacy and safety. Additionally, strategies to improve the tolerability of tirzepatide may enhance its practical application. This includes patient education, dosing titration to minimize side effects, and investigating novel therapies that offer similar or greater effectiveness and safety.

## Conclusion

This meta-analysis demonstrated the efficacy of tirzepatide in reducing body weight, BMI, WC, HbA1c, blood pressure, and improving quality of life in ﻿overweight or obese adults without diabetes. However, the increased risk of adverse events warrants assessment of benefits versus risks of tirzepatide before its use, careful monitoring and close follow-up of therapy. This study highlights the need for continued research directly comparing various medications to fully elucidate the therapeutic role of tirzepatide in obesity or overweight management.

## Supplementary Information

Below is the link to the electronic supplementary material.

Supplementary file 1
