# Supplementary material for: Efficacy and safety of tirzepatide versus placebo in overweight or obese adults without diabetes: a systematic review and meta-analysis of randomized controlled trials
Source: Int J Clin Pharm. 2024 Jul 22;46(6):1268–80. doi: 10.1007/s11096-024-01779-x (PMC11576767; doi:10.1007/s11096-024-01779-x)
Supplement: Supplementary file 1 [file 11096_2024_1779_MOESM1_ESM.docx]

**Supplementary file 1. Search strategy and key terms**

(tirzepatide or LY3298176 or "dual GIP and GLP-1RA" or " dual glucagon-like peptide and glucose-dependent insulinotropic polypeptide" or Zepbound) AND (Random or RCT or RCTs or "randomized controlled trial" or trial* or "clinical trial*")

**PubMed:**

| Query | Results |
| --- | --- |
| Search: (tirzepatide or LY3298176 or "dual GIP and GLP-1RA" or " dual glucagon-like peptide and glucose-dependent insulinotropic polypeptide" or Zepbound) AND (Random or RCT or RCTs or "randomized controlled trial" or trial* or "clinical trial*") | 200 |

Embase:

| No | Query | Results | Date |
| --- | --- | --- | --- |
| #1 | ('tirzepatide'/exp OR tirzepatide OR 'ly3298176'/exp OR ly3298176 OR 'dual gip and glp-1ra' OR 'dual glucagon-like peptide and glucose-dependent insulinotropic polypeptide' OR zepbound) AND (random OR rct OR rcts OR 'randomized controlled trial'/exp OR 'randomized controlled trial' OR trial* OR 'clinical trial*') | 144 | 18-Jan-24 |

**The Cochrane Library:**

259 Trials matching (tirzepatide or LY3298176 or "dual GIP and GLP-1RA" or " dual glucagon-like peptide and glucose-dependent insulinotropic polypeptide" or Zepbound) AND (Random or RCT or RCTs or "randomized controlled trial" or trial* or "clinical trial*") in Title Abstract Keyword - (Word variations have been searched)

Fig. S1

1) 5% weight loss


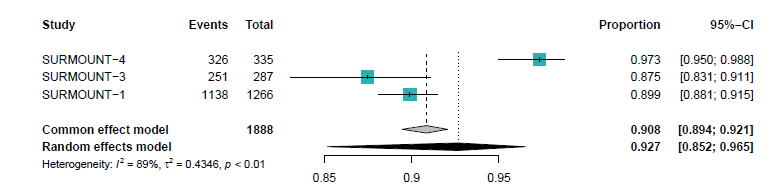


2) 10% weight loss


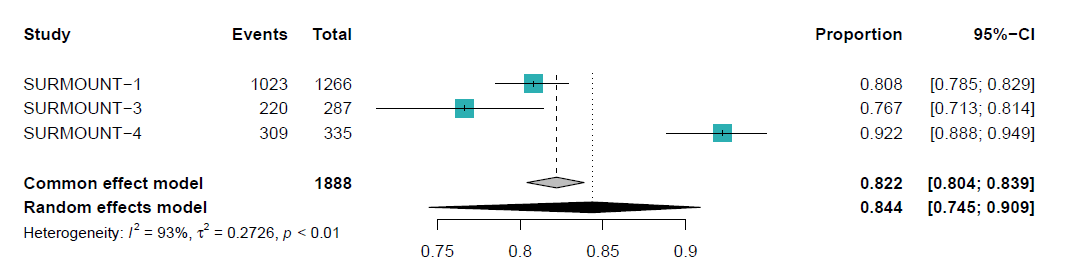


3) 15% weight loss


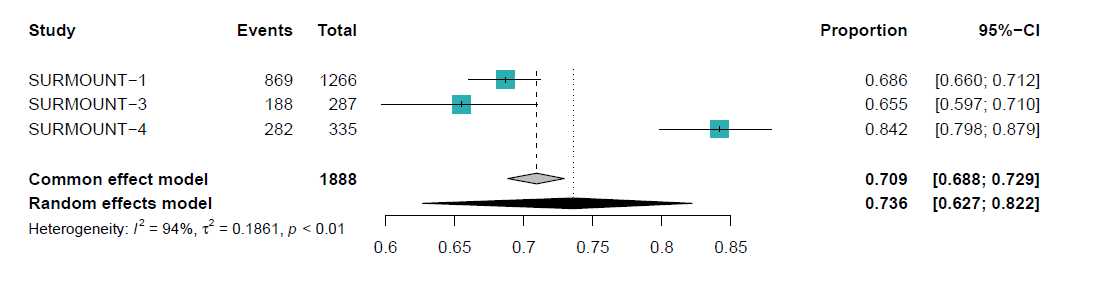


4) 20% weight loss


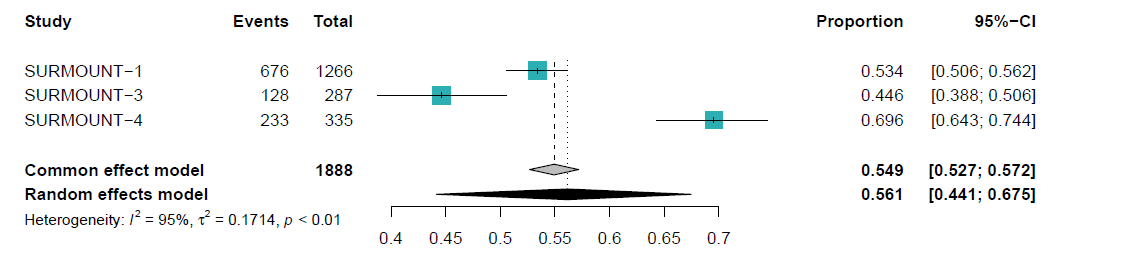


5) 25% weight loss


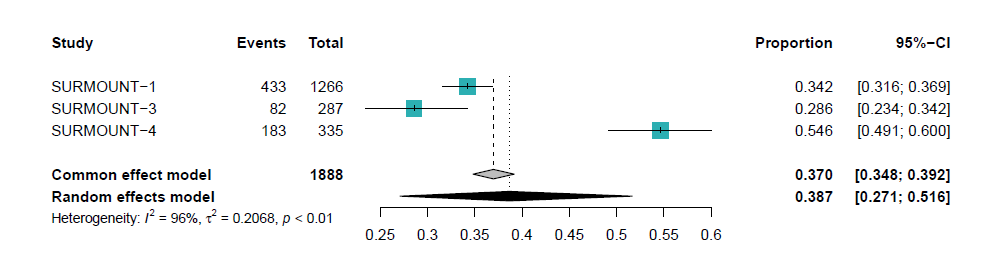


6) Weight loss (%)


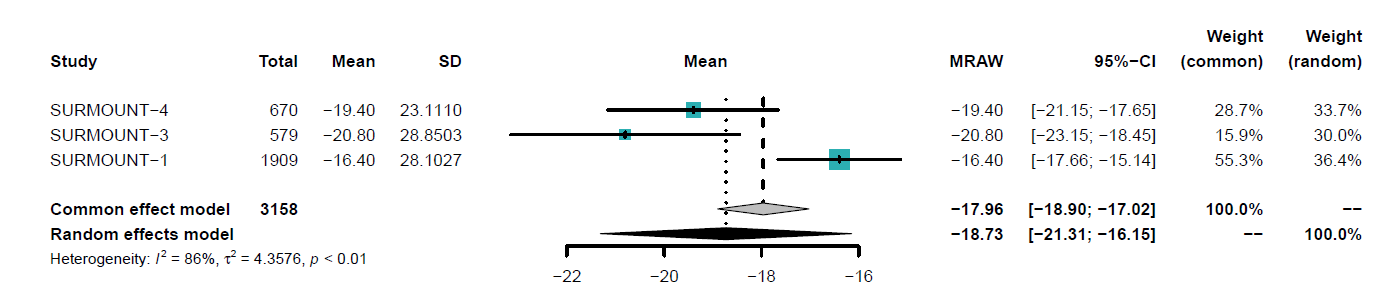


7) BMI (kg/m^2^)


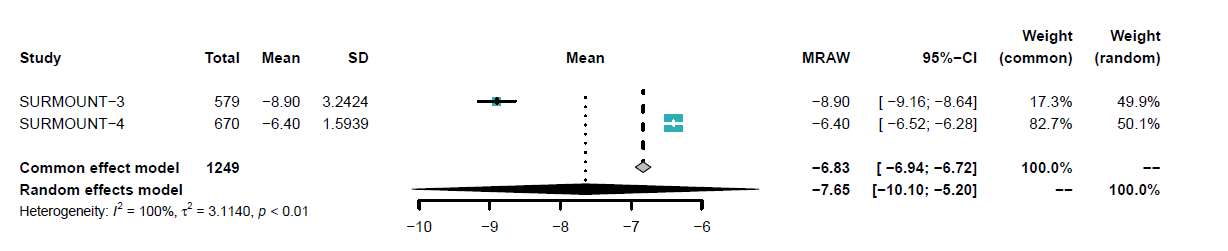
8) Waist circumference (cm)


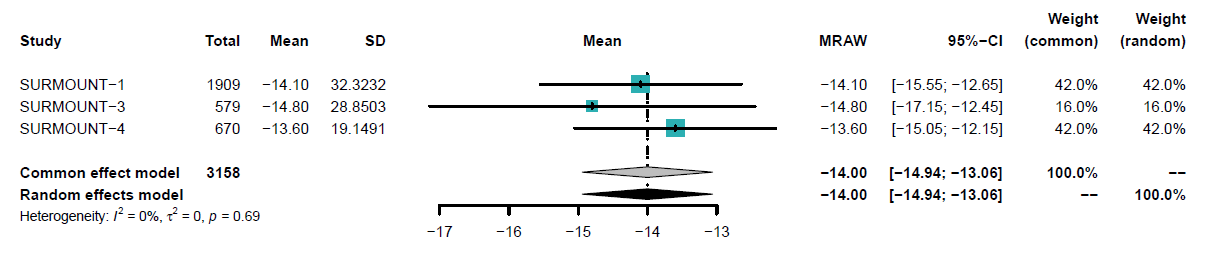


9) HbA1c (%)


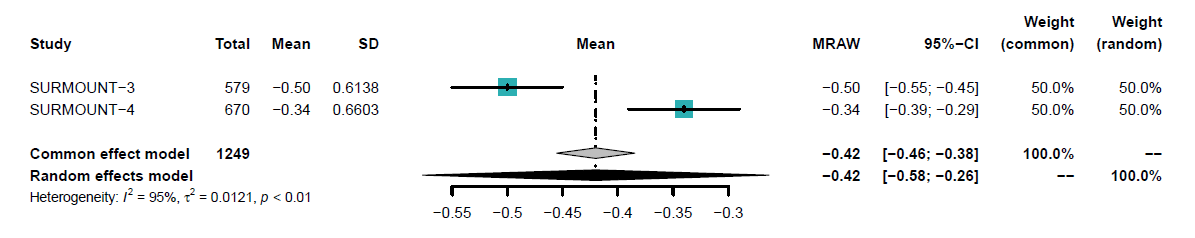


10) SBP (mmHg)
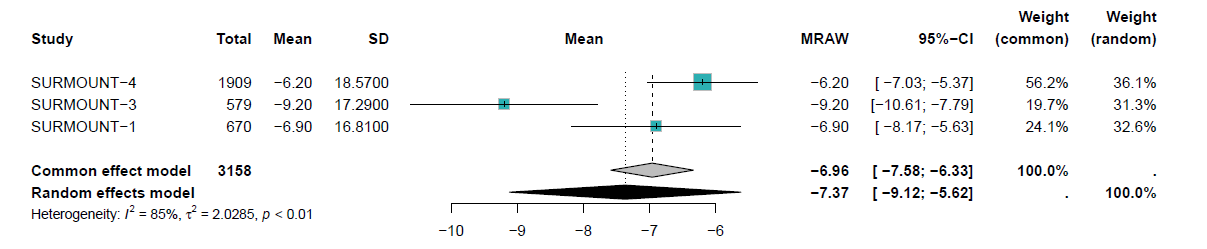
 11) DBP (mmHg)
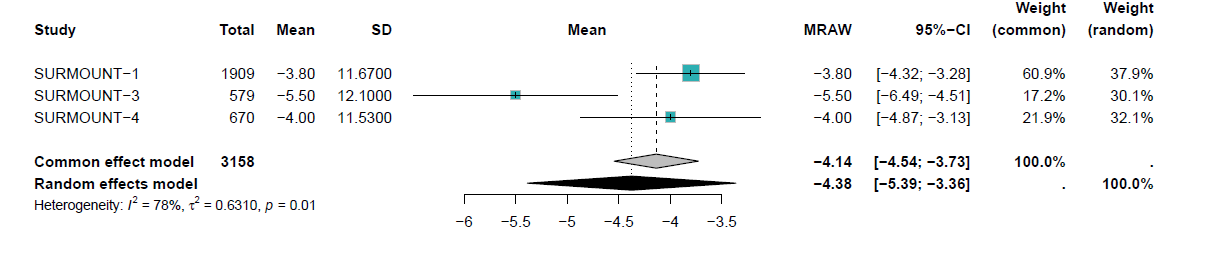


12) SF-36 PF score


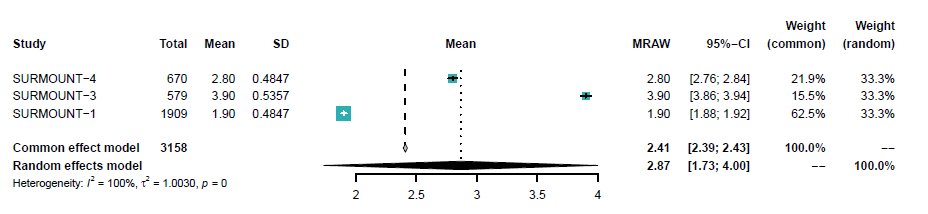


13) IWQOL score


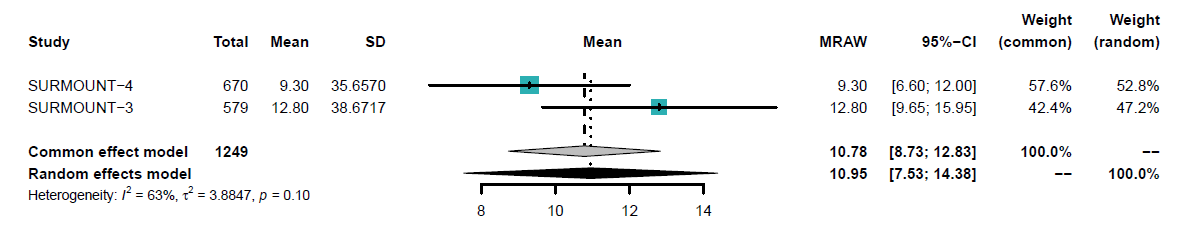


Pooled efficacy results of tirzepatide versus placebo: 1) 5% weight loss; 2) 10% weight loss; 3) 15% weight loss; 4) 20% weight loss; 5) 25% weight loss; 6) Weight loss (%); 7) BMI (kg/m2); 8) Waist circumference (cm); 9) HbA1c (%); 10) SBP (mmHg); 11) DBP (mmHg); 12) SF-36 PF score; 13) IWQOL score. Abbreviations: BMI: body mass index, SBP: systolic blood pressure; DBP: diastolic blood pressure, HbA1c: hemoglobin A1c, SF-36 PF: short form-36 health survey physical functioning domain, IWQOL: impact of weight on quality of life, CI: confidence interval; MRAW: combined means.

Fig. S2

1) HbA1c (%)


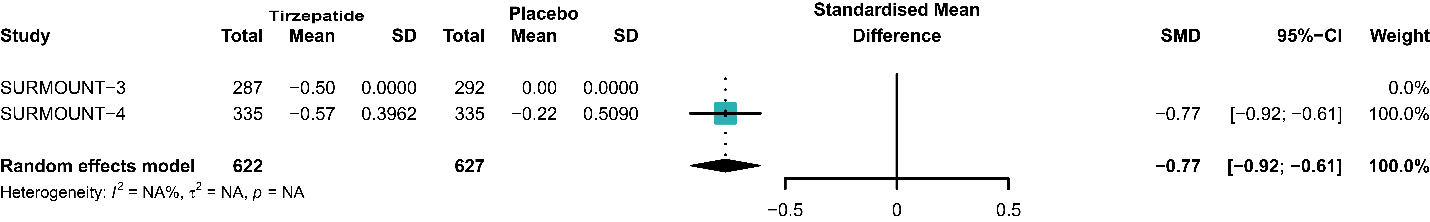


2) SBP (mmHg)


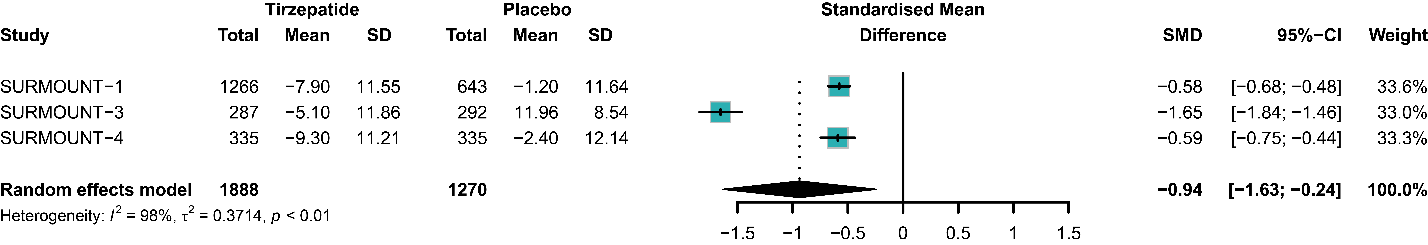


3) DBP (mmHg)


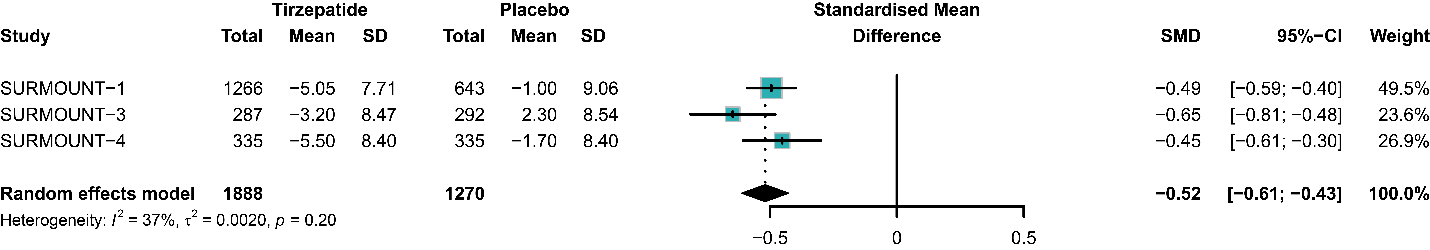


4) IWQOL score


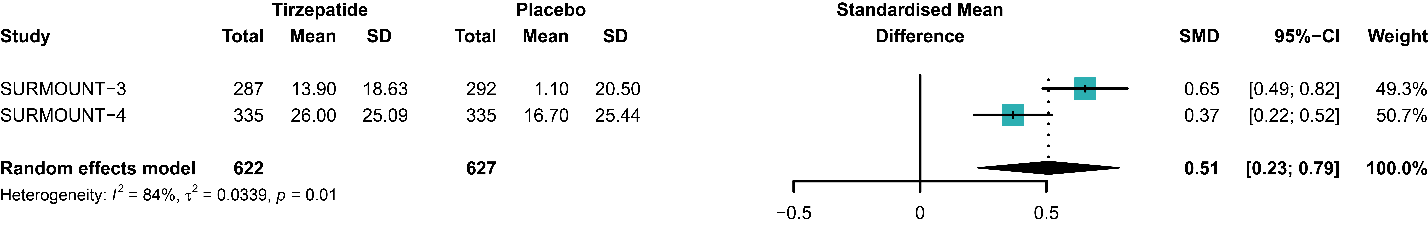


5) SF-36 PF score


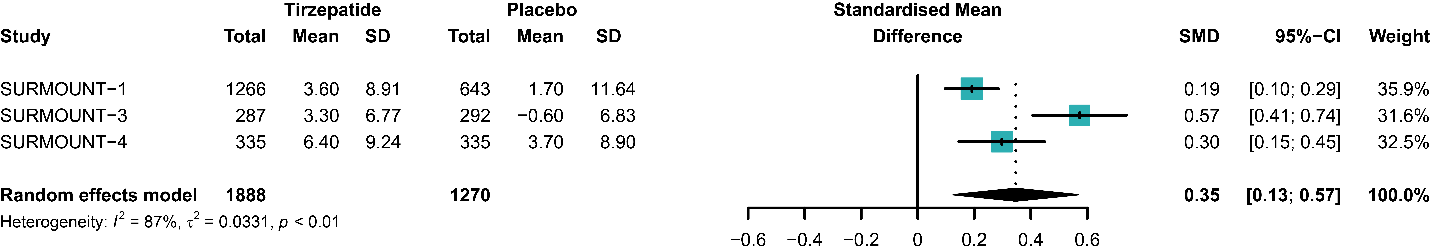


Comparison of tirzepatide versus placebo for 1) HbA1c, 2) SBP, 3) DBP, 4) IWQOL score, 5) SF-36 PF score. Abbreviations: SF-36 PF: short form-36 health survey physical functioning domain, IWQOL: impact of weight on quality of life, SBP: systolic blood pressure; DBP: diastolic blood pressure; HbA1c: hemoglobin A1c, SMD: standard mean deviation, CI: confidence interval.

Table S1. The risk of bias assessment of included studies.

| Study | Year | Bias arising from randomization process | Bias due to deviations from intended interventions | Bias due to missing outcome data | Bias in measurement of the outcome | Bias in selection of the reported result | Overall |
| --- | --- | --- | --- | --- | --- | --- | --- |
| SURMOUNT 1 | 2022 | + | + | + | + | + | + |
| SURMOUNT 3 | 2023 | + | + | + | + | + | + |
| SURMOUNT 4 | 2024 | + | + | + | + | + | + |

| + |
| --- |

Low risk of bias
